# Alpha synuclein co-pathology is associated with accelerated amyloid-driven tau accumulation in Alzheimer’s disease

**DOI:** 10.1186/s13024-025-00822-3

**Published:** 2025-03-18

**Authors:** Nicolai Franzmeier, Sebastian Niclas Roemer-Cassiano, Alexander Maximilian Bernhardt, Amir Dehsarvi, Anna Dewenter, Anna Steward, Davina Biel, Lukas Frontzkowski, Zeyu Zhu, Johannes Gnörich, Julia Pescoller, Fabian Wagner, Fabian Hirsch, Hannah de Bruin, Rik Ossenkoppele, Carla Palleis, Felix Strübing, Michael Schöll, Johannes Levin, Matthias Brendel, Günter U. Höglinger

**Affiliations:** 1 Institute for Stroke and Dementia Research (ISD), University Hospital, LMU Munich, Munich, Germany; 2 Munich Cluster for Systems Neurology (SyNergy), Munich, Germany; 3 The Sahlgrenska Academy, Institute of Neuroscience and Physiology, Department of Psychiatry and Neurochemistry, University of Gothenburg, Mölndal and Gothenburg, Sweden; 4 Department of Neurology, LMU University Hospital, University Hospital, LMU Munich, Munich, Germany; 5 German Center for Neurodegenerative Diseases (DZNE), Munich, Germany; 6 Department of Nuclear Medicine, University Hospital, LMU Munich, Munich, Germany; 7 Alzheimer Center Amsterdam, Neurology, Vrije Universiteit Amsterdam, Amsterdam, Netherlands; 8 Amsterdam Neuroscience, Neurodegeneration, Amsterdam, Netherlands; 9 Clinical Memory Research Unit, Lund University, Lund, Sweden; 10 Center for Neuropathology and Prion Research, University Hospital, LMU Munich, Munich, Germany; 11 Wallenberg Centre for Molecular and Translational Medicine, University of Gothenburg, Gothenburg, Sweden; 12 Dementia Research Centre, Queen Square Institute of Neurology, University College London, London, UK; 13 Department of Neuropsychiatry, Sahlgrenska University Hospital, Gothenburg, Sweden

## Abstract

**Background:**

Aggregated alpha-Synuclein (αSyn) is a hallmark pathology in Parkinson’s disease but also one of the most common co-pathologies in Alzheimer’s disease (AD). Preclinical studies suggest that αSyn can exacerbate tau aggregation, implying that αSyn co-pathology may specifically contribute to the Aβ-induced aggregation of tau that drives neurodegeneration and cognitive decline in AD. To investigate this, we combined a novel CSF-based seed-amplification assay (SAA) to determine αSyn positivity with amyloid- and tau-PET neuroimaging in a large cohort ranging from cognitively normal individuals to those with dementia, examining whether αSyn co-pathology accelerates Aβ-driven tau accumulation and cognitive decline.

**Methods:**

In 284 Aβ-positive and 308 Aβ-negative subjects, we employed amyloid-PET, Flortaucipir tau-PET, and a CSF-based αSyn seed-amplification assay (SAA) to detect in vivo αSyn aggregation. CSF p-tau_181_ measures were available for 384 subjects to assess earliest tau abnormalities. A subset of 155 Aβ-positive and 135 Aβ-negative subjects underwent longitudinal tau-PET over approximately 2.5 years. Using linear regression models, we analyzed whether αSyn SAA positivity was linked to stronger Aβ-related increases in baseline fluid and PET tau biomarkers, faster Aβ-driven tau-PET increase, and more rapid cognitive decline.

**Results:**

αSyn SAA positivity was more common in Aβ + vs. Aβ- subjects and increased with clinical severity (*p* < 0.001). Most importantly, αSyn positivity was also associated with greater amyloid-associated CSF p-tau_181_ increases (*p* = 0.005) and higher tau-PET levels in AD-typical brain regions (*p* = 0.006). Longitudinal analyses confirmed further that αSyn positivity was associated with faster amyloid-related tau accumulation (*p* = 0.029) and accelerated amyloid-related cognitive decline, potentially driven driven by stronger tau pathology.

**Conclusions:**

Our findings suggest that αSyn co-pathology, detectable via CSF-based SAAs, is more prevalent in advanced AD and contributes to the development of aggregated tau pathology thereby driving faster cognitive decline. This highlights that a-Syn co-pathology may specifically accelerate amyloid-driven tau pathophysiology in AD, underscoring the need to consider αSyn in AD research and treatment strategies.

## Introduction

Alzheimer’s disease (AD) is neuropathologically defined by the presence of amyloid-beta (Aβ) plaques and neurofibrillary tau tangles [1]. The development of AD is currently understood as a cascade of pathophysiological events, where initial Aβ aggregation is assumed to trigger the subsequent trans-neuronal spread of tau pathology, ensuing neurodegeneration and cognitive decline [2–5]. However, *post mortem* studies have shown that the AD-defining pathologies Aβ and tau are rarely found in isolation, but that concomitance of proteinopathies, including TDP-43, Lewy body pathology and cerebral amyloid angiopathy is the rule rather than the exception [1, 6–9]. The frequency of co-pathologies increases with age, with the large majority of AD patients showing at least one co-pathology at autopsy, questioning the existence of pure AD pathology especially in older individuals, but also in autosomal dominant AD [1, 8–10]. Therefore, it is crucial to understand how these molecular co-pathologies interact with the core AD pathologies Aβ and tau and how they contribute to disease progression. Lewy body pathology consisting of alpha-synuclein (αSyn) aggregates, i.e. the hallmark pathology of Parkinson’s disease and Lewy body dementia [11], is one of the most common co-pathologies found in ~ 50% of AD cases at autopsy [6, 7, 9] and concomitant αSyn has been shown to exacerbate cerebral glucose hypometabolism, cognitive impairment and rate of decline in AD patients [7, 12–14]. *Post mortem* studies have further shown that the degree of αSyn co-pathology in AD is related to the severity of both Aβ and tau burden [8], suggesting a close link between AD and αSyn co-pathology.

Previous research has reported that αSyn may interact with tau pathology in AD. Specifically, tau and αSyn are both intracellular proteins that are highly abundant in neurons [15, 16], and αSyn has been shown to be capable of inducing tau hyperphosphorylation [17–20], i.e., one of the earliest hallmarks of pathophysiological tau alterations in AD [21]. Further, both proteins have been shown to mutually promote each other’s fibrilization in vitro and in vivo [22–25] and to co-aggregate in neurons and their synaptic terminals [23, 26, 27]. Recent studies in PS19 mice have further shown that αSyn specifically accelerates the spreading of tau pathology, whereas the presence of tau pathology did not promote the progression of αSyn aggregates [27, 28]. In addition, the knock-down of αSyn reduced the effect of αSyn seeds on the appearance and spread of tau pathology [27], together suggesting that αSyn co-pathology may promote tau aggregation and spread in AD, thereby contributing to neurodegeneration and faster cognitive decline.

However, this question could not be addressed in AD patients previously, since reliable in vivo biomarkers of αSyn were not readily available [29]. Recently, advances in biomarker development have demonstrated that αSyn pathology can be assessed in patients based on cerebrospinal fluid (CSF) seed amplification assays (SAA), that quantify the seeding and self-replicative aggregation potential of αSyn seeds present in the CSF of patients [30]. These αSyn SAAs can identify patients with αSyn pathology of the Lewy-type (e.g., Parkinson’s disease, Lewy body dementia) already in prodromal disease stages and discriminate those Synucleinopathies from non-αSyn-related diseases [31, 32]. Previous research in AD patients has shown that ante-mortem performed CSF-based αSyn SAAs have a particularly high sensitivity and specificity in detecting cortical rather than focal subcortical αSyn co-pathology in patients with AD post-mortem [33, 34], which is potentially relevant for a physical interaction between αSyn and tau, given the primary cortical appearance and spreading of tau in AD [35–38]. Studies employing αSyn SAAs in AD patient cohorts have confirmed higher rates of αSyn co-pathology in patients with an AD-typical biomarker profile and have reported synergistic effects of αSyn and AD pathology on cognitive decline [33, 39–41], where αSyn SAA positive individuals show earlier cognitive decline after onset of amyloidosis [42]. These findings therefore support previous *post mortem* evidence of αSyn co-pathology contributing to clinical severity in AD [7–9, 33]. However, it is still unclear whether αSyn co-pathology may specifically promote tau pathology in AD patients and thereby contribute to disease progression.

Thus, in the current study, we examined (i) whether the presence of αSyn co-pathology as assessed via CSF-based SAAs promotes tau pathophysiology and aggregation in an AD context, and (ii) whether the putative αSyn-associated acceleration of tau aggregation contributes to faster cognitive decline. To achieve this, we analyzed data from 592 subjects ranging from cognitively normal to dementia from the Alzheimer’s Disease Neuroimaging Initiative (ADNI), with available cross-sectional amyloid-PET and tau-PET data to assess fibrillar Aβ and tau pathology, as well as CSF-based αSyn SAA data to identify αSyn pathology. In this dataset, we tested whether αSyn co-pathology was linked to increased tau-PET tracer signal in the context of abnormal Aβ. Additionally, in a subset of 384 subjects with CSF p-tau_181_ data, we explored whether αSyn co-pathology was associated with elevated tau hyperphosphorylation and p-tau release from neurons [43, 44], a process that may specifically precede and drive tau aggregation [45]. Lastly, we utilized longitudinal tau-PET and longitudinal cognitive data from a subset of 290 subjects to determine whether αSyn co-pathology specifically accelerates Aβ-related tau aggregation rates and cognitive decline over time in AD patients.

## Methods

### Sample

592 individuals were included from the Alzheimer’s Disease Neuroimaging Initiative (ADNI) database, based on availability of clinical data, baseline 18 F-Florbetapir/Florbetaben amyloid-PET, 3T MRI and 18 F-Flortaucipir tau-PET data. Individuals with major neurological diseases than AD or severe psychiatric conditions that may affect cognition are excluded from ADNI. A detailed overview of ADNI inclusion and exclusion criteria can be found online (https://adni.loni.usc.edu/wp-content/themes/freshnews-dev-v2/documents/clinical/ADNI3_Protocol.pdf). All baseline data had to be obtained within a timeframe of 6 months, in line with our previous studies using ADNI data [46–50]. This time window was chosen to maximize observations with complete neuroimaging and clinical data within a given timeframe. Participants diagnostic status was determined by ADNI as cognitively normal (CN; Mini Mental State Examination [MMSE] ≥ 24, Clinical Dementia Rating [CDR] = 0, non-depressed), mildly cognitively impaired (MCI; MMSE ≥ 24, CDR = 0.5, objective memory-impairment on education-adjusted Wechsler Memory Scale II, preserved activities of daily living) and demented (MMSE = 20–26, CDR ≥ 0.5, National Institute of Neurological and Communicative Disorders and Stroke/Alzheimer’s Disease and Related Disorders Association criteria for probable AD). 410 out of the total sample of 592 subjects also had CSF measures of p-tau_181_ available to determine earliest tau pathophysiology. P-tau_181_ values were adjusted to Aβ_1−40_ levels, which are considered to be relatively stable and serve as a reference protein, following a previous suggestion that the p-tau_181_/Aβ_1−40_ ratio is a better measure of tau pathophysiology than p-tau_181_ alone [51]. All CSF measures were determined by the ADNI biomarker core on the Roche Elecsys Cobas e 601 platform (for a methods description, please see https://adni.bitbucket.io/reference/docs/UPENNBIOMK12_2020/ADNI_UPENNBIOMK12_Methods_2020_Roche_Elecsys_ADNI3_CSFs.pdf). A subset of 290 subjects had longitudinal 18 F-Flortaucipir data available to model longitudinal tau accumulation. Amyloid status was determined on global amyloid-PET SUVRs using tracer-specific cut-offs (i.e. Aβ + = SUVR > 1.11/1.08 for Florbetapir/Florbetaben) previously established in the ADNI cohort [52, 53]. All study procedures were conducted in accordance with the declaration of Helsinki, ethical approval was obtained by ADNI investigators. All study participants provided written informed consent.

#### αSyn seed amplification assay

The αSyn SAAs were performed as described previously [33] in the Amprion Clinical Laboratory (CLIA ID No. 05D2209417; CAP No. 8168002) using a method validated for clinical use in accordance with Clinical Laboratory Improvement Amendment (CLIA) requirements. All αSyn SAA data were provided by the ADNI biomarker core. Each sample was analyzed by Amprion in triplicate in a 96-well plate using a reaction mixture comprised of 100mM PIPES pH 6.5, 0.5 M NaCl, 0.1% sarkosyl, 10µM ThT, 0.3 mg/mL recombinant αSyn, and 40µL CSF, in a final volume of 100µL. Two silicon nitride beads are included in each well, and positive and negative assay quality control samples are included on each plate. Plates are sealed with optical adhesive film, placed into the chamber of a BMG LABTECH FLUOstar Ω Microplate Reader, and incubated at 42 °C with cycles of 1 min of shaking followed by 14 min of rest with fluorescence measured after every shaking cycle (excitation wavelength 440 nm, emission 490 nm). After a total incubation time of 20 h, the maximum fluorescence for each well is determined and an algorithm applied to the triplicate determinations for each sample for result classification. Samples were considered positive when all triplicates showed seeding activity and negative if no seeding activity was observed. Undetermined samples in which only a subset of the three triplicates was positive were excluded from the study.

#### Neuroimaging acquisition

Structural MRI was acquired on 3T scanners. T1-weighted structural scans were collected using an MPRAGE sequence (TR = 2300ms; Voxel size = 1 × 1 × 1 mm; for parameter details see: https://adni.loni.usc.edu/wp-content/uploads/2017/07/ADNI3-MRI-protocols.pdf). PET data was assessed using post intravenous injection of ^18^F-labeled tracers (Flortaucipir: 6 × 5 min time-frames, 75–105 min post-injection; Florbetapir: 4 × 5 min time-frames, 50–70 min post-injection; Florbetaben: 4 × 5 min time-frames, 90–110 min post-injection; for more information see http://adni.loni.usc.edu/methods/pet-analysis-method/pet-analysis/).

#### Image processing

All images were screened for artifacts before preprocessing. T1-weighted structural MRI scans were bias-corrected, segmented, and non-linearly warped to Montreal Neurological Institute (MNI) space using the CAT12 toolbox (https://neuro-jena.github.io/cat12-help/). Harmonized PET images were downloaded from the ADNI loni database (https://ida.loni.usc.edu/). The harmonization has been implemented by the ADNI PET core (described online https://adni.loni.usc.edu/data-samples/adni-data/neuroimaging/pet/) and includes realignment of dynamic PET scans as well as averaging to obtain single Flortaucipir/Florbetapir image. For harmonization, all images are smoothed to a common resolution using scanner-specific 3D-Gaussian filters derived by the University of Michigan team using Hoffman phantom scans carried out at each site. The effective resolution was selected by the ADNI PET core based on the lowest resolution scanners in ADNI (i.e. 8 mm Full width at half maximum). Resulting downloaded images have a standardized voxel size and spatial resolution. These images were rigidly registered to the T1-weighted MRI scan. Reference regions (i.e., inferior cerebellar grey for Flortaucipir, whole cerebellum for Florbetapir/Florbetaben) [54] and the cortical Schaefer atlas including 200 regions of interest (ROIs) were warped from MNI to T1-native space using the CAT12-derived non-linear normalization parameters, masked with subject-specific grey matter and applied to PET data to determine standardized uptake value ratios (SUVRs) for each region of the Schaefer 200 atlas [55]. Global and regional Florbetapir/Florbetaben SUVRs were converted to centiloid using equations provided by ADNI.

#### Statistical analysis

All statistical analyses were performed in R (Version 4.3.1). Patient characteristics for the cross-sectional and longitudinal datasets were compared between groups stratified by amyloid and diagnostic status (i.e. CN, MCI, Dementia) using Chi-squared tests for nominal and ANOVAs for continuous measures. Logistic regression was further used to determine αSyn SAA positivity differences between diagnostic groups while additionally adjusting for age as a confound. To determine longitudinal tau-PET changes (i.e., for the 200 Schaefer ROIs, Braak-stage specific ROIs and temporal meta-ROI) and cognitive change rates (i.e., for the ADAS13), we employed linear mixed models controlling for random slope and intercept as described previously [37, 38].

To address our main aims, linear regression analyses were performed to determine whether the αSyn SAA status modulated the effect of global amyloid-PET centiloid levels on (i) the p-tau_181_/Aβ_40_ ratio (i.e., αSyn SAA x centiloid interaction), (ii) tau-PET in the temporal meta ROI, (iii) tau-PET change rates in the temporal meta ROI. Exploratory analyses were conducted for Braak-stage specific tau-PET ROIs and 200 ROIs of the Schaefer atlas (FDR-corrected to account for 200 regions). To determine whether αSyn SAA status modulated AD-related cognitive decline via elevated tau pathology we further determined the (iv) αSyn SAA x centiloid or (v) αSyn SAA x temporal meta tau-PET interaction on ADAS13 change rates. Exploratory sub-analyses were conducted using MMSE scores. Regression models were controlled for age, sex and study site for cross-sectional analyses, and additionally for follow-up years for analyses using longitudinal data. For our main five analyses described above, we applied FDR-correction to account for multiple testing (correcting for five tests). For confirmatory purposes, we further performed ANCOVAs to determine main effects of αSyn SAA status on tau-PET SUVRs, stratified by amyloid-PET status. ANCOVA models were controlled for age, sex, study site and centiloid levels.

## Results

**Fig. 1:**
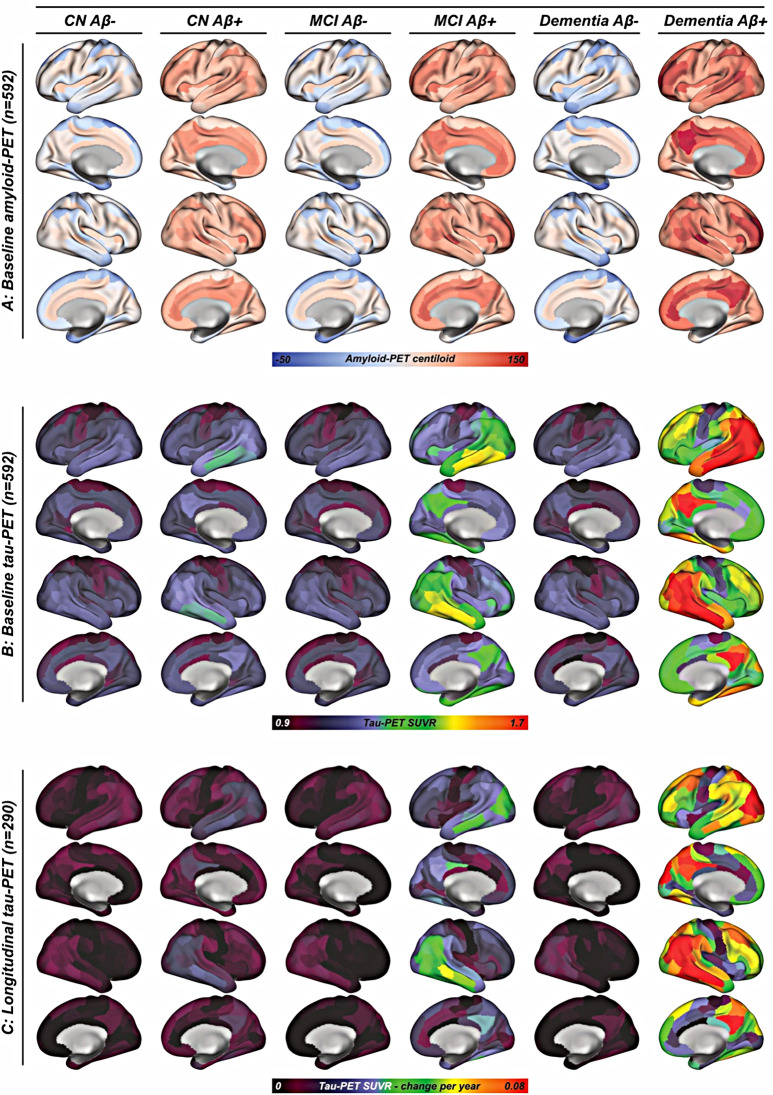
Surface renderings of (**A**) baseline amyloid-PET centiloids and (**B**) tau-PET baseline as well as (**C**) annualized tau-PET rates of change, stratified by diagnostic group and amyloid-PET status at baseline

**Fig. 2:**
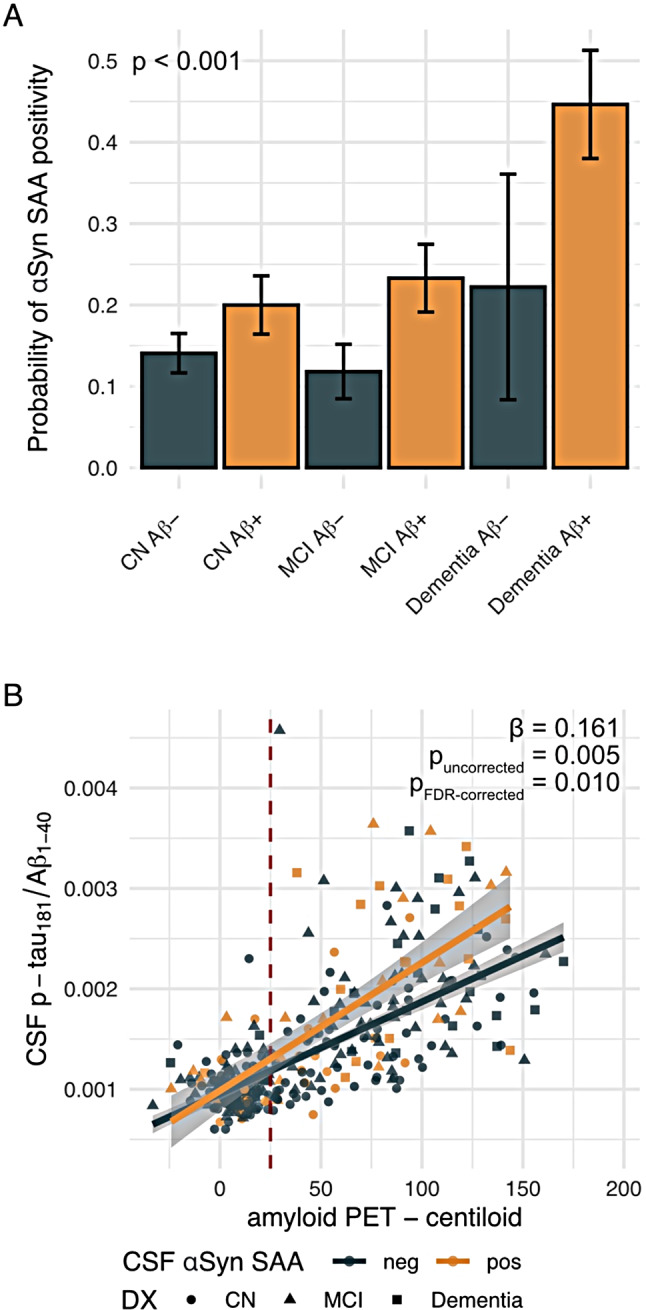
Bar plot illustrating the probability of αSyn SAA positivity stratified by amyloid status and clinical syndrome severity. Statistical significance was determined via logistic regression controlling for age (**A**). Scatterplot illustrating the interaction effect between amyloid-PET and αSyn SAA status on cross-sectional levels of CSF p-tau_181_/Aβ_1 − 40_ as an indicator of earliest tau pathophysiology. Diagnostic groups are indicated by shape, the cut-point of amyloid-PET positivity is indicated by the dashed red line at 25 centiloids. The beta value indicates the strength of the amyloid-PET x CSF αSyn SAA interaction effect as determined by linear regression, controlling for age, sex and study site. FDR-correction for multiple comparisons (*p* < 0.05) was applied for the main analyses (**B**), adjusting for five statistical tests

We included 331 cognitively normal (CN) individuals, 196 patients with mild cognitive impairment (MCI) and 65 patients with clinically diagnosed dementia all with available baseline amyloid-PET (i.e., [^18^F]florbetaben/[^18^F]florbetapir, *n* = 197/395), [^18^F]flortaucipir tau-PET and CSF-based αSyn data based on a pre-established SAA (Amprion, San Diego, CA) to determine the αSyn status [30]. All amyloid-PET SUVR data was transformed to centiloids to harmonize across the two amyloid-PET tracers [56]. A subset of 384 subjects had cross-sectional CSF p-tau_181_ levels measured on the Roche Elecsys platform for assessing earliest tau pathophysiology which were referenced to CSF Aβ_1 − 40_ levels to adjust for overall CSF protein concentrations [51]. Further, 290 subjects (CN/MCI/dementia = 178/84/28) had longitudinal flortaucipir tau-PET and cognitive assessments over 2.9 ± 1.4 years. Baseline amyloid-PET centiloids and tau-PET SUVRs as well as longitudinal tau-PET change rates stratified by Aβ and diagnostic status are shown in Fig. 1, illustrating AD-typical temporo-parietal tau-PET uptake with increased clinical disease severity in the Aβ-positive (Aβ+) groups, while no tau-PET increase was found in Aβ-negative (Aβ-) groups. Sample demographics, biomarker and clinical data are shown in Table 1 for both the cross-sectional (*N* = 592) and longitudinal samples (*N* = 290). Cross-sectionally, we found that the prevalence of αSyn positivity increased with more severe clinical impairment and was particularly pronounced in Aβ+ (i.e., CN/MCI/dementia = 20/23/45%) vs. Aβ- subjects (i.e., CN/MCI/dementia = 14/12/22%, chi-squared-test, *p* < 0.001, Fig. 2A). This result was consistent when additionally controlling for age in a logistic regression model (*p* < 0.001), suggesting that αSyn co-pathology is more common in clinically advanced AD, regardless of age. No difference in amyloid-PET centiloids was found between SAA-positive vs. negative subjects for Ab+ (F = 0.162, *p* = 0.688) and Ab- subjects (F = 2.424, *p* = 0.113), controlling for age, sex and site.

**Table 1:** Sample characteristics

**Cross-sectional (***n* = **592)**	**CN Aβ-(***n* = **206)**	**CN Aβ+(***n* = **125)**	**MCI Aβ-(***n* = **93)**	**MCI Aβ+(***n* = **103)**	**Dementia Aβ-(***n* = **9)**	**Dementia Aβ+(***n* = **56)**	**p-value**
Age	72.47 (6.87)	75.56 (7.46)	72.68 (8.83)	74.98 (7.23)	74.84 (7.56)	77.14 (9.40)	< 0.001
Sex female (%)	119 (57.8)	79 (63.2)	39 (41.9)	44 (42.7)	3 (33.3)	24 (42.9)	0.002
Sex male (%)	87 (42.2)	46 (36.8)	54 (58.1)	59 (57.3)	6 (66.7)	32 (57.1)	
CSF p-tau181 pg/ml (SD)^1^	18.56 (6.80)	28.90 (13.56)	19.68 (7.72)	35.27 (18.89)	19.06 (7.14)	35.98 (13.62)	< 0.001
Amyloid-PET centiloid (SD)	7.46 (8.45)	63.97 (33.34)	5.82 (11.47)	79.26 (33.23)	4.48 (13.20)	99.75 (36.15)	< 0.001
Temporal meta Tau-PET SUVR (SD)	1.14 (0.10)	1.20 (0.14)	1.13 (0.09)	1.41 (0.33)	1.16 (0.11)	1.70 (0.47)	< 0.001
ADAS13 Total (SD)	7.99 (4.39)	8.20 (4.86)	11.17 (4.37)	17.00 (6.40)	25.73 (2.78)	31.46 (8.19)	< 0.001
MMSE (SD)	29.09 (1.16)	28.86 (1.54)	28.98 (1.21)	27.35 (2.46)	24.40 (4.04)	21.61 (4.97)	< 0.001
αSyn SAA neg (%)	177 (85.9)	100 (80.0)	82 (88.2)	79 (76.7)	7 (77.8)	31 (55.4)	< 0.001
αSyn SAA pos (%)	29 (14.1)	25 (20.0)	11 (11.8)	24 (23.3)	2 (22.2)	25 (44.6)	
**Longitudinal** **(n = 290)**	**CN Aβ-** **(n = 99)**	**CN Aβ+** **(n = 79)**	**MCI Aβ-** **(n = 31)**	**MCI Aβ+** **(n = 53)**	**Dementia Aβ-** **(n = 5)**	**Dementia Aβ+** **(n = 23)**	**p-value**
Age	72.06 (6.60)	75.13 (6.78)	71.86 (8.64)	73.79 (6.55)	75.02 (5.19)	74.88 (8.95)	0.051
Sex female (%)	58 (58.6)	52 (65.8)	13 (41.9)	24 (45.3)	1 (20.0)	10 (43.5)	0.034
Sex male (%)	41 (41.4)	27 (34.2)	18 (58.1)	29 (54.7)	4 (80.0)	13 (56.5)	
CSF p-tau181 (SD)	19.11 (7.05)	27.37 (12.21)	17.46 (8.58)	36.48 (21.11)	17.70 (7.70)	34.18 (13.27)	< 0.001
Amyloid-PET centiloid (SD)	7.28 (7.94)	67.68 (35.58)	5.50 (12.69)	77.62 (30.95)	-2.73 (13.62)	98.88 (31.24)	< 0.001
Temporal meta Tau-PET SUVR (SD)	1.15 (0.12)	1.21 (0.13)	1.14 (0.08)	1.40 (0.33)	1.14 (0.05)	1.66 (0.40)	< 0.001
Temporal meta Tau-PET SUVR annual change rate (SD)	0.01 (0.01)	0.01 (0.02)	0.00 (0.01)	0.04 (0.04)	0.00 (0.00)	0.07 (0.05)	< 0.001
Tau-PET follow-up years	3.52 (1.38)	2.76 (1.38)	2.76 (1.47)	2.52 (1.19)	1.47 (0.56)	1.77 (0.67)	< 0.001
ADAS13 Total (SD)	7.61 (4.24)	8.20 (4.86)	11.17 (4.37)	17.00 (6.40)	25.73 (2.78)	31.46 (8.19)	< 0.001
MMSE (SD)	29.19 (0.89)	28.70 (1.69)	29.20 (1.15)	27.40 (2.28)	25.00 (1.41)	21.10 (3.96)	< 0.001
αSyn SAA neg (%)	88 (88.9)	63 (79.7)	28 (90.3)	40 (75.5)	4 (80.0)	13 (56.5)	0.007
αSyn SAA pos (%)	11 (11.1)	16 (20.3)	3 (9.7)	13 (24.5)	1 (20.0)	10 (43.5)	

### αSyn SAA positivity is linked to elevated p-tau in the context of Ab

We first tested whether SAA positivity was associated with changes in the earliest signs of tau pathophysiology in AD, i.e., increases of hyperphosphorylated p-tau in CSF that typically precede tau tangle formation. This analysis was performed in 384 subjects with available cross-sectional p-tau_181_/Aβ_1 − 40_ data. We found a significant SAA-positivity status-by-centiloid interaction effect on the p-tau_181_/Aβ_1 − 40_ ratio when controlling for age, sex and study site, showing that the p-tau_181_/Aβ_1 − 40_ increase in response to Aβ deposition was stronger in SAA–positive subjects compared to SAA-negative subjects (*β* = 0.161, *p*_*uncorrected*_=0.005, *p*_*FDR−corrected*_=0.010, Fig. 2). This result was consistent when controlling for diagnosis (*β* = 0.126, *p* = 0.0246) or when considering only p-tau_181_ instead of the p-tau_181_/Aβ_1 − 40_ ratio as a dependent variable (*β* = 0.11, *p* = 0.035), while additionally controlling for Aβ_1 − 40_ levels. Additionally controlling for vascular co-pathology (WMH volume) in a subset of 136 subjects, yielded largely consistent results (*β* = 0.196, *p* = 0.065). Together, these results suggest that αSyn SAA positivity is associated with stronger p-tau_181_ release in response to Aβ pathology.

### αSyn SAA positivity is associated with higher tau-PET tracer signal in the context of AD pathology

**Fig. 3:**
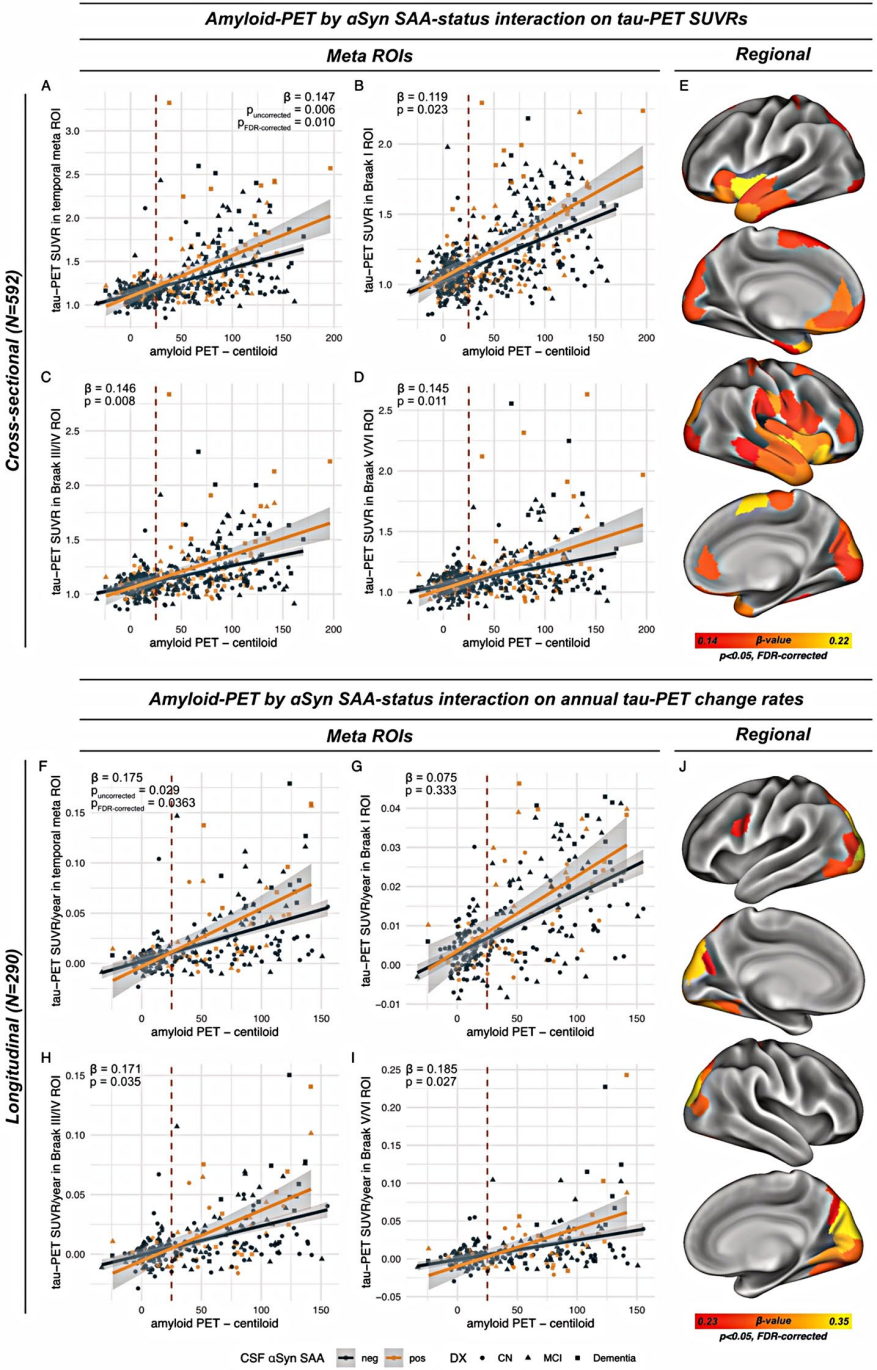
Scatterplots illustrating the interaction effect between global amyloid-PET (i.e. centiloid) and aSyn SAA status on tau-PET SUVRs for meta-ROIs (**A**-**D**) and regional analyses (**E**), as well as for annual tau-PET SUVR change rates (**F**-**J**). Diagnostic groups are indicated by shape, the cut-point of amyloid-PET positivity is indicated by the dashed red line at 25 centiloids. Beta values indicate the strength of the amyloid-PET x CSF αSyn SAA interaction effect, as determined by linear regression, controlling for age, sex and study site (A-E) as well as maximum tau-PET follow-up time (**F**-**J**). FDR-correction for multiple comparisons (*p* < 0.05) was applied for the main analyses (**A**, **F**), adjusting for five statistical tests. In addition, ROI-wise assessments were also FDR-corrected at *p* < 0.05

**Fig. 4:**
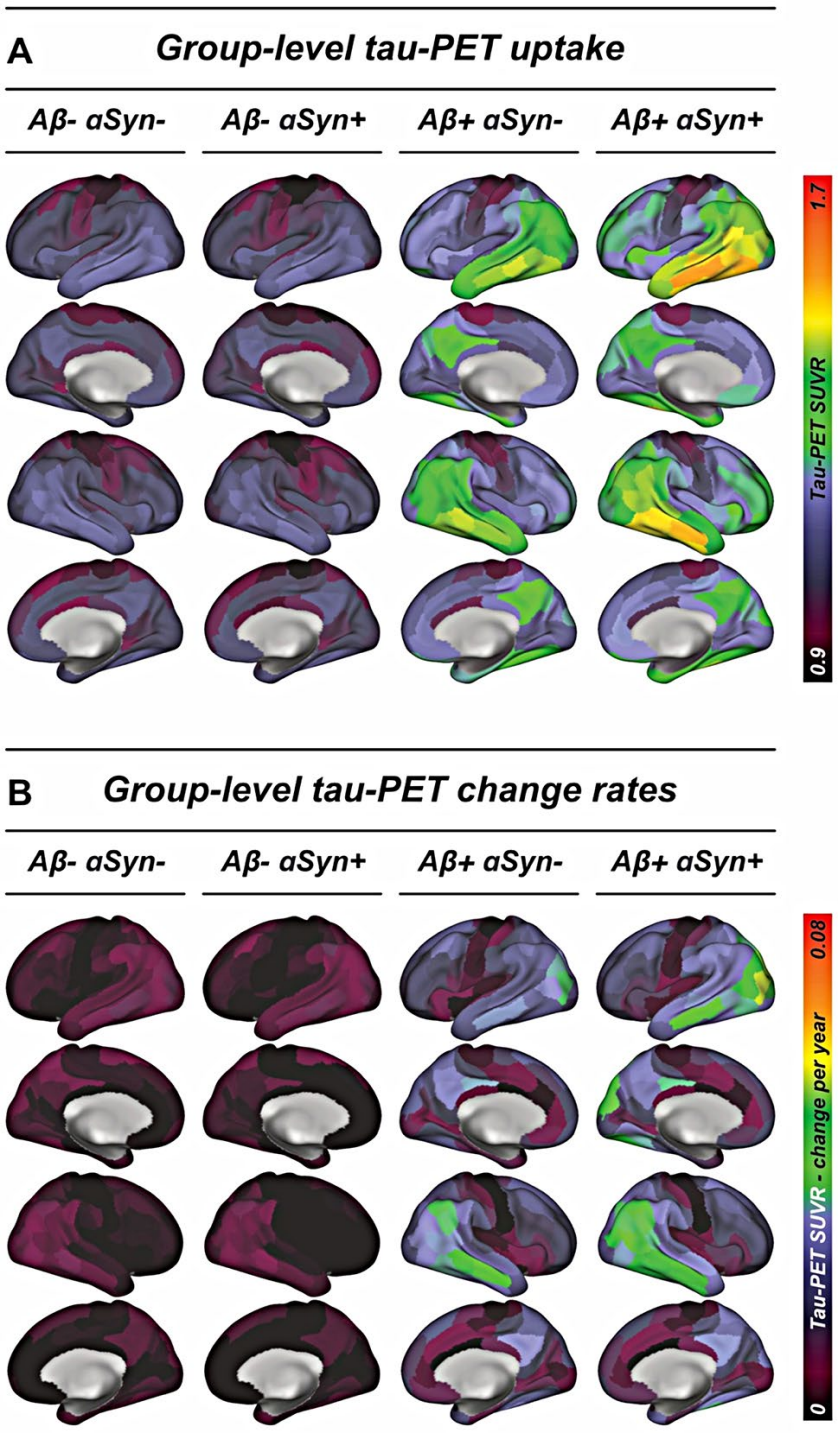
Surface renderings of tau-PET uptake (**A**) and annual tau-PET change rates (**B**) stratified by amyloid-PET and αSyn SAA status

Next, we investigated whether SAA positivity was linked to stronger tau-PET tracer signal in an AD context, given that αSyn seeds have been proposed exacerbate tau aggregation [22–27]. Supporting this view, we found a significant interaction effect between amyloid-PET centiloid levels and SAA positivity on tau-PET SUVRs in the temporal meta-ROI that captures AD-typical tau accumulation, controlling for age, sex and study site (*β* = 0.147, *p*_*uncorrected*_=0.006, *p*_*FDR−corrected*_=0.010). As shown in Fig. 3A, SAA-positive individuals showed a greater increase of tau-PET SUVRs at higher amyloid-PET levels compared to SAA-negative individuals at similar amyloid-PET levels. An exploratory extension of this analysis to the pre-defined Braak-stage regions that capture tau progression [48] yielded consistent results for the centiloid-by-SAA-interaction across Braak_I_ (*β* = 0.119, *p* = 0.0226, Fig. 3B), Braak_III/IV_ (*β* = 0.146, *p* = 0.008, Fig. 3C) and Braak_V/VI_ ROIs (*β* = 0.144, *p* = 0.017, Fig. 3D), indicating that SAA-positivity-related aggravation of tau deposition in an AD context not confined to a particularly vulnerable brain region. Results remained largely consistent when additionally controlling for diagnosis, except for Braak_I_ (temporal meta/Braak_I/III−IV/V−VI_, *β* = 0.104/0.079/0.112/0.120, *p* = 0.045/0.116/0.041/0.035). This was further substantiated by ROI-based analyses, showing widespread SAA-by-centiloid interaction effects predominantly on temporo-frontal tau-PET uptake (Fig. 3E). To further confirm that SAA positivity is specifically related to higher tau in the presence of abnormal Aβ levels, we additionally tested for a main effect of SAA positivity on tau-PET SUVRs stratified by Aβ status, using ANCOVAs including the covariates age, sex, study site and centiloid levels (tau-PET SUVRs stratified by SAA and amyloid status are shown in Fig. 4A). Here, we found that SAA positivity was associated with increased temporal meta tau-PET SUVRs in Aβ + subjects (*p* = 0.008) but not in Aβ- subjects (*p* = 0.889). Consistent results were detected for regional analyses in Braak-stage-specific ROIs for Braak_I_ (Aβ+/-, *p* = 0.004/0.369), Braak_III/IV_ (Aβ+/-, *p* = 0.008/0.961) and Braak_V/VI_ (Aβ+/-, *p* = 0.021/0.521). Together, these findings suggest that SAA positivity is associated with increased Aβ-associated tau deposition in individuals on the AD spectrum.

### αSyn SAA positivity is associated with faster Aβ-related Tau accumulation

Next, we tested whether SAA positivity was not only linked to higher cross-sectional tau-PET levels in the presence of Aβ, but also to faster tau accumulation rates, indicative of accelerated Aβ-related tau seeding and spread. To this end, we used linear regression models to test the SAA-status-by-centiloid-interaction on annual tau-PET change rates, using the subset of 290 subjects with longitudinal flortaucipir tau-PET data. Congruent with the cross-sectional results, we found that SAA positivity was related to faster Aβ-related tau-PET change rates in the temporal meta-ROI (*β* = 0.175, *p*_*uncorrected*_=0.029, *p*_*FDR−corrected*_=0.0363, Fig. 3F). Exploratorily extending this analysis to Braak-stage specific regions showed faster Aβ-related tau accumulation in Braak_III/IV_ (*β* = 0.171, *p* = 0.035, Fig. 3H) and Braak_V/VI_ (*β* = 0.185, *p* = 0.027, Fig. 3I) but not in Braak_I_ (*β* = 0.075, *p* = 0.333, Fig. 3G). Again, these results were largely consistent when additionally controlling for diagnosis (temporal meta/Braak_I/III−IV/V−VI_, *β* = 0.160/0.045/0.167/0.172, *p* = 0.039/0.551/0.040/0.042). Confirming that the effects of SAA positivity on accelerated tau accumulation were driven by subjects on the AD spectrum (i.e. Aβ+), we found that SAA positivity had a significant main effect on faster tau-PET change rates in the temporal meta-ROI in the Aβ+ (*p* = 0.037) but not in the Aβ- subjects (*p* = 0.899), controlling for age, sex, study site and centiloid levels. This result pattern was congruent for Braak_I_ (Aβ+/Aβ-, *p* = 0.047/0.795), Braak_IV/V_ (Aβ+/Aβ-, *p* = 0.039/0.883) and trend-level for Braak_V/VI_ (Aβ+/Aβ-, *p* = 0.071/0.891). Together, these findings support the view that SAA positivity is related to faster Aβ-related accumulation of fibrillar tau pathology over time in subjects on the AD spectrum.

### αSyn SAA positivity is associated with accelerated Aβ-related cognitive decline

**Fig. 5:**
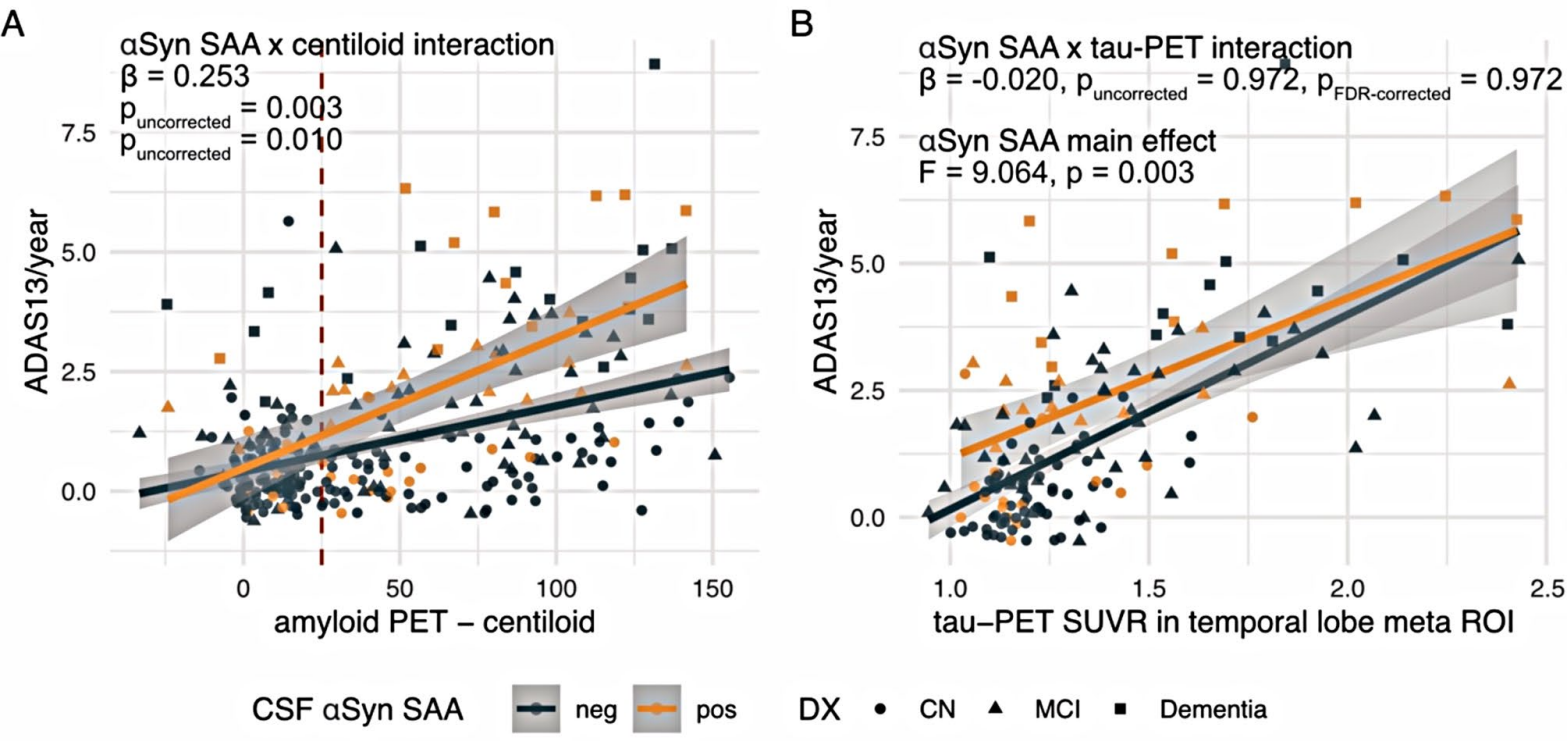
Scatterplot illustrating the effect of αSyn SAA status on the association between amyloid-PET centiloid (**A**) and tau-PET (**B**) at baseline on subsequent change rates in the ADAS13 score of global cognition. Diagnostic groups are indicated by shape, the cut-point of amyloid-PET positivity is indicated by the dashed red line at 25 centiloids. Beta values indicate the strength of the amyloid-PET x CSF αSyn SAA interaction effect (**A**) or the tau-PET x CSF αSyn SAA interaction effect (**B**), as determined by linear regression, controlling for age, sex and study site and maximum follow up time of cognitive assessments. FDR-correction for multiple comparisons (*p* < 0.05) was applied for the main regression analyses, adjusting for five statistical tests

Lastly, we tested whether LB co-pathology was associated with accelerated AD-related cognitive decline. Supporting this, we found a significant centiloid-by SAA-αSyn-status-interaction on annual change rates in ADAS13 global cognitive scores (*β* = 0.253, *p*_*uncorrected*_=0.003, *p*_*FDR−corrected*_=0.010, Fig. 5A), controlling for age, sex and study site. This result remained consistent when additionally controlling for diagnosis (*β* = 0.132, *p* = 0.0246) or tau-PET in the temporal meta ROI (*β* = 0.153, *p* = 0.030), or when using annual change rates in the MMSE score as an alternative measure of cognitive decline (*β*=-0.194, *p* = 0.039, available for a subset of *N* = 247 individuals). In contrast, no interaction between temporal meta tau-PET SUVRs and αSyn SAA positivity was found on ADAS13 change rates (*β*=-0.020, *p*_*uncorrected*_=0. 0.972, *p*_*FDR−corrected*_=0.972, Fig. 5B), controlling for age, sex and centiloid levels. However, there was a main effect of αSyn positivity on ADAS13 change rates (*p* = 0.003; controlling for age, sex, temporal meta-ROI tau-PET and centiloid), suggesting that SAA positivity is related to overall faster cognitive decline. This effect was present at lower tau-PET levels and vanished at higher tau-PET levels. Taken together, this suggests that SAA positivity is related to faster cognitive decline in AD, and that this may be particularly driven by higher SAA-positivity-related tau-PET burden in individuals with high Aβ burden.

## Discussion

The major aim of this study was to investigate whether αSyn Lewy-body pathology, one of the most common co-pathologies found *post-mortem* in AD patients [6, 7, 9], accelerates the development of tau pathology in AD. This was motivated by previous evidence from animal and in vitro studies suggesting that αSyn aggregates exacerbate tau pathophysiology, promoting its hyperphosphorylation [18, 20], fibrillization and spread, thereby potentially contributing to AD progression [27, 28]. Consistent with previous cohort studies [33, 39], we observed that positive αSyn seeding activity in the CSF, indicative of diffuse cortical αSyn co-pathology [33, 34], was more frequent in individuals across the AD spectrum (i.e., amyloid-PET positive) compared to controls, particularly in those with more advanced clinical impairment. Our first main finding was that αSyn SAA positivity was associated with elevated Aβ-related CSF p-tau_181_ levels, suggesting enhanced tau hyperphosphorylation and secretion of p-tau in response to Aβ in AD patients with evidence of Lewy-body co-pathology. Second, we found that αSyn SAA positivity was linked to stronger Aβ-associated fibrillar tau deposition in AD-typical brain regions, as assessed by flortaucipir tau-PET imaging. This was further substantiated by longitudinal tau-PET analyses, showing that αSyn SAA positivity at baseline was related to faster rates of Aβ-driven tau accumulation in AD typical brain regions, overall supporting the hypothesis that αSyn co-pathology exacerbates the Aβ-driven formation of AD-type neurofibrillary tau pathology. Third, we found that αSyn co-pathology was related to faster AD-related cognitive decline, which was potentially driven by faster tau accumulation in αSyn SAA positive individuals on the AD spectrum (i.e. amyloid-PET positive). Collectively, these findings suggest that αSyn co-pathology, as detected via CSF-based αSyn SAAs, is associated with an acceleration of Aβ-induced tau pathophysiology across the AD spectrum ensuing faster cognitive decline, overall positioning αSyn as a significant modulator of tauopathy in the context of Aβ pathology, as well as clinical disease progression in AD.

Congruent with previous neuropathological studies highlighting a prevalence of up to 50% of αSyn co-pathology in AD patients [6, 7, 9], we found increased αSyn SAA positivity rates in individuals across the AD spectrum (i.e., Aβ+) compared to controls (i.e., Aβ-), with αSyn SAA positivity rates rising alongside clinical severity up to 44% in AD dementia. This finding aligns with previous results from the larger overall ADNI CSF database (*N* ~ 1600) [33, 42], and with data from European cohorts, reporting increased αSyn SAA positivity across the AD spectrum with 36–45% αSyn SAA positivity rates in AD dementia patients [41, 57]. Therefore, our results converge with *post mortem* neuropathological evidence [6, 7, 9], pinpointing to αSyn as a key and highly prevalent molecular co-pathology throughout the AD spectrum that can be detected via CSF-based αSyn SAAs.

Addressing our key goal to determine the role of αSyn co-pathology in AD-related tau pathophysiology, our first major finding was that Aβ-associated CSF p-tau_181_ levels were higher in αSyn SAA positive vs. negative individuals. CSF p-tau increases have previously been shown to be more closely linked to PET-assessed fibrillar Aβ than to fibrillar tau pathology [58] and to reflect Aβ-associated active secretion of newly synthesized p-tau molecules, rather than passive spill out from dead neurons [43, 59]. In this context, it is possible that αSyn co-pathology may strengthen the Aβ-driven neuronal release of hyperphosphorylated p-tau in AD. Tau is released from neurons in an activity-dependent manner, e.g., via entering synaptic vesicles [60–62], and Aβ has been found to trigger aberrant neuronal hyperactivity [63, 64], e.g., by impairing glutamate re-uptake [65] and causing decreased GABA sensitivity [66]. Pathomechanistically, Aβ-induced neuronal hyperactivity may therefore promote the synaptic release of p-tau seeds that enter downstream neurons, inducing template-based tau misfolding and aggregation, ensuing a cascade of tau spread across interconnected neurons [45, 61]. Supporting this, we showed recently that higher Aβ triggers neuronal hyperconnectivity, thereby promoting faster tau spread [67]. αSyn is typically located in presynaptic terminals and has been shown to co-regulate the activity-dependent release of neurotransmitters, e.g., via modulating the assembly of SNARE complexes that are key mediators of presynaptic vesicle fusion and neurotransmitter release [68–71]. Supporting a role of αSyn in neuronal activity, studies in transgenic mice expressing human mutant αSyn found aberrant network hyperexcitability [72, 73], and extracellular αSyn oligomers have been shown to trigger excessive astrocytic glutamate release, which may further drive aberrant neuronal activity and neuronal p-tau release [74]. Yet again, other studies have suggested that pathologically altered αSyn disrupts synaptic signaling and attenuates neuronal activity [75, 76]. Given these opposing findings, the exact role of αSyn in modulating the activity-dependent neuronal release of p-tau in the presence of Aβ deposition remains to be determined. An alternative explanation for our findings is that αSyn may accelerate intra-neuronal tau phosphorylation as shown by several pre-clinical studies [17–20], suggesting hyperphosphorylated tau seeds being released from neurons. Preliminary evidence along those lines suggests slight increases in CSF and plasma p-tau_181_ biomarker levels in Parkinson’s disease patients when compared to controls [77, 78]. Yet, the exact mechanisms of how αSyn co-pathology may exacerbate the Aβ-related secretion of p-tau in the context of neuronal activity changes remains to be determined in future studies, where our findings can act as a key starting point to motivate this research.

Our second major finding was that αSyn SAA positivity was associated with stronger Aβ-related aggregation of cortical tau pathology, as shown by elevated Aβ-related tau-PET tracer binding in αSyn SAA positive vs. negative individuals. This association was widespread and found consistently throughout brain regions corresponding to Braak stages I-VI which are commonly used to determine the spatial distribution of tau pathology in AD [35, 48, 79]. This cross-sectional result was further substantiated by longitudinal tau-PET analyses, showing faster Aβ-related tau accumulation in αSyn SAA positive individuals throughout key tau vulnerable brain regions beyond the medial temporal lobe. These results support our central hypothesis that αSyn co-pathology may facilitate the Aβ-driven aggregation and spread of tau in AD and thereby contribute to the progression of AD pathophysiology. Our results are in agreement with previous findings from animal and cell culture models, showing that the induction of pre-formed αSyn fibrils can act as a co-seeding factor for tau, promoting its aggregation into fibrillar forms [23, 24, 27], while knockdown of endogenous αSyn attenuated tau aggregation and spread [27]. Additionally, as described above, αSyn pathology can enhance tau phosphorylation (e.g. via activating the tau specific kinase GSK-3β), thereby potentially rendering tau more aggregation-prone [18]. Further, higher CSF p-tau levels are linked to faster trans-neuronal spread of tau pathology [45], hence the αSyn-pathology related CSF p-tau secretion may also contribute to accelerated tau aggregation and spread. Therefore, αSyn co-pathology may accelerate tau aggregation through both direct interactions such as co-seeding as well as kinase-mediated hyperphosphorylation pathways or increased p-tau secretion ensuing faster tau spreading [23, 24, 27, 45, 80]. Our results are of significant clinical relevance, as tau pathology is highly prognostic of future neurodegeneration [81] and cognitive decline in AD [48, 82]. Results from a successful recent phase 3 anti-Aβ trial have shown further that the clinical efficacy of monoclonal anti-Aβ antibodies is limited by the presence of advanced fibrillar tau pathology [83]. More specifically, the removal of Aβ may no longer yield clinical benefits once tau pathology has accumulated to a substantial degree where it enters a self-driving auto-aggregation cycle that is detached from Aβ pathology [45, 84]. Since our results have shown that αSyn co-pathology accelerates the Aβ-associated aggregation of tau pathology in AD, the presence of αSyn co-pathology may be a limiting factor for anti-Aβ drugs to prevent downstream tau aggregation.

Lastly, we found that CSF αSyn SAA positivity is linked to faster AD-related cognitive decline. This finding recapitulates previous *post mortem* evidence that αSyn co-pathology contributes to the overall cognitive burden in AD patients [7, 12, 13] and similarly, previous longitudinal cohort studies have shown that CSF αSyn SAA positive patients with an AD biomarker profile show faster cognitive decline, regardless of clinical status [39, 40, 42]. Here, more recent findings from the ADNI cohort could show that αSyn SAA positive individuals show earlier decline in cognition after the onset of amyloidosis, and that cognitive decline accelerates in individuals who convert from αSyn SAA negative to positive after conversion [42]. However, we found only a synergistic effect between αSyn and amyloid-PET, but not between αSyn and tau-PET levels, on faster cognitive decline. Since we found αSyn SAA positivity to be related to faster Aβ-related tau accumulation, this result pattern suggests that αSyn co-pathology accelerates cognitive deterioration in AD primarily by accelerating the Aβ-related aggregation of fibrillar tau pathology and associated symptom worsening. Nevertheless, we also detected an independent main effect of αSyn SAA positivity next to tau pathology on cognitive decline, suggesting that individuals with concomitant αSyn pathology show generally stronger cognitive deficits at any given level of tau severity. Importantly, we only investigated changes in global cognition, and did not specifically assess symptoms that are more specific for αSyn pathology, such as hallucinations, fluctuations in attention and alertness, or motor dysfunction. Here, previous studies have emphasized that αSyn SAA positive AD patients show more pronounced αSyn-related symptoms on top of AD-related cognitive deficits [40], supporting the view that αSyn co-pathology is associated with a more complex clinical syndrome characterized by mixed AD and αSyn-related symptomatology [85]. This finding of concomitant αSyn co-pathology affecting cognition is potentially relevant for patient-level prognosis and clinical trials, which should optimally factor in αSyn pathology as a contributor to cognitive deterioration, next to other key co-pathologies such as small-vessel disease [86] that accelerate cognitive decline in older individuals.

While the overall results provide a converging picture of αSyn contributing to Aβ-related tau pathophysiology and cognitive decline across the AD spectrum, several limitations should be considered when interpreting the results of the current study. First, CSF-based αSyn SAAs determine the presence or absence of αSyn co-pathology as a binary measure, based on seeding activity of αSyn in the CSF [31]. However, αSyn co-pathology is most likely a continuum ranging from low to high αSyn burden, and quantitation of αSyn severity using SAAs is not yet established. Therefore, we cannot determine whether more severe αSyn co-pathology further increases the effect of Aβ on tau accumulation. In addition, a synergistic effect of αSyn on promoting tau aggregation potentially requires co-localization and close interaction between both proteins. This view is supported by in vitro and animal studies [24, 28, 87] and potentially showcased by a case report of an atypical AD patient with strongly lateralized and co-localized αSyn and tau deposition at autopsy. Since CSF-based αSyn SAAs do not encode any spatial information about the pattern of αSyn co-pathology, we can, however, not determine whether local αSyn promotes co-localized tau accumulation until reliable PET tracers for αSyn become available. Investigating the spatial relationship between αSyn deposits and tau aggregation and spread will therefore be a major endeavor for future PET investigations on the role of αSyn co-pathology in AD pathophysiology once αSyn tracers are established. However, CSF-based αSyn SAAs typically show highest sensitivity for detecting diffuse cortical rather than focal subcortical or brainstem αSyn co-pathology in AD, as shown by post-mortem assessments [33, 34]. Therefore, a positive αSyn SAA result likely indicates diffuse cortical αSyn co-pathology, thereby increasing the likelihood of αSyn co-pathology promoting the cortical aggregation and spread of tau. Nevertheless, protein-protein interactions cannot be mapped in living humans, hence the exact mechanisms by which αSyn and tau interact to promote tau aggregation and spread remain to be investigated by studies employing in vitro or animal model systems. In addition, the current study did not include longitudinal CSF p-tau_181_ data due to limited availability, therefore these results remain to be confirmed by future studies investigating whether aSyn co-pathology drives faster and earlier p-tau increases in the context of Aβ pathology. Lastly, we determined the impact of aSyn co-pathology on global cognition using ADAS13 or MMSE assessments. Yet, αSyn co-pathology in AD may manifest in motor symptoms beyond AD-typical cognitive manifestations. Since standardized assessment of Parkinsonian sypmtoms (e.g. UPDRS) is not part of the clinical ADNI workflows, addressing this remains to be determined by future studies.

In conclusion, our findings suggest that αSyn co-pathology, as detected via CSF-based αSyn SAAs, plays a critical role in accelerating Aβ-induced tau pathophysiology and cognitive decline across the AD spectrum. Our findings are a critical extension of previous pre-clinical and in vitro investigations on the interaction between Aβ, tau and αSyn, overall confirming the idea that αSyn co-pathology may actively modulate the amyloid cascade and accelerate the transition from amyloidosis to tauopathy. These findings have implications for disease prognostication, since αSyn co-pathology could be considered as a factor that may additionally promote tau aggregation and spread, thereby contributing to the development of AD dementia. Yet, the contribution of αSyn SAA positivity to individualized disease prognostication models should be investigated in future studies. Together, our results should motivate further research, assessing the exact mechanisms by which αSyn may accelerate the Aβ-tau axis, which could help us understand how to prevent the transition from amyloidosis to tauopathy in AD and potentially establish αSyn co-pathology as a treatment target in AD.

## Data Availability

The data that support the findings of this study are available from the ADNI database (adni.loni.usc.edu) upon registration and compliance with the data use agreement.

## Abbreviations

AD: Alzheimer’s Disease
Aβ: Amyloid-beta
αSyn: Alpha-Synuclein
SAA: Seed Amplification Assay
PET: Positron Emission Tomography
CSF: Cerebrospinal Fluid
p-tau181: Phosphorylated tau at threonine 181
SUVR: Standardized Uptake Value Ratio
MMSE: Mini-Mental State Examination
CDR: Clinical Dementia Rating
MCI: Mild Cognitive Impairment
CN: Cognitively Normal
MRI: Magnetic Resonance Imaging
ADNI: Alzheimer’s Disease Neuroimaging Initiative
GSK-3β: Glycogen Synthase Kinase 3 Beta
